# Emergence and Spread of Extended Spectrum β-Lactamase Producing Enterobacteriaceae (ESBL-PE) in Pigs and Exposed Workers: A Multicentre Comparative Study between Cameroon and South Africa

**DOI:** 10.3390/pathogens8010010

**Published:** 2019-01-16

**Authors:** Luria Leslie Founou, Raspail Carrel Founou, Noyise Ntshobeni, Usha Govinden, Linda Antoinette Bester, Hafizah Yousuf Chenia, Cyrille Finyom Djoko, Sabiha Yusuf Essack

**Affiliations:** 1 Antimicrobial Research Unit, School of Health Sciences, College of Health Sciences, University of KwaZulu-Natal, Durban 4000, South Africa; czangue@yahoo.fr (R.C.F.); Govindenu@ukzn.ac.za (U.G.); Essacks@ukzn.ac.za (S.Y.E.); 2 Department of Food Safety and Environmental Microbiology, Centre of Expertise and Biological Diagnostic of Cameroon (CEDBCAM), Yaoundé 8242, Cameroon; 3 Department of Clinical Microbiology, Centre of Expertise and Biological Diagnostic of Cameroon (CEDBCAM), Yaoundé 8242, Cameroon; 4 Discipline of Microbiology School of Life Sciences, College of Agriculture, Engineering and Sciences, University of KwaZulu-Natal, Durban 4000, South Africa; nb.ntshobeni@gmail.com (N.N.); Cheniah@ukzn.ac.za (H.Y.C.); 5 Biomedical Resource Unit, School of Laboratory Medicine and Medical Sciences, College of Health Sciences, University of KwaZulu-Natal, Durban 4000, South Africa; Besterl@ukzn.ac.za; 6 Centre for Research and Doctoral Training in Life Science, Health and Environment, The Biotechnology Centre, University of Yaoundé I, Yaoundé 337, Cameroon; cfdjoko@gmail.com; 7 Metabiota Inc., Yaoundé 8242, Cameroon

**Keywords:** antibiotic resistance, Enterobacteriaceae, ESBL, food chain, one health

## Abstract

Extended spectrum β-lactamase-producing *Enterobacteriaceae* (ESBL-PE) represent a significant public health concern globally and are recognized by the World Health Organization as pathogens of critical priority. However, the prevalence of ESBL-PE in food animals and humans across the farm-to-plate continuum is yet to be elucidated in Sub-Saharan countries including Cameroon and South Africa. This work sought to determine the risk factors, carriage, antimicrobial resistance profiles and genetic relatedness of extended spectrum β-lactamase producing *Enterobacteriaceae* (ESBL-PE) amid pigs and abattoir workers in Cameroon and South Africa. ESBL-PE from pooled samples of 432 pigs and nasal and hand swabs of 82 humans were confirmed with VITEK 2 system. Genomic fingerprinting was performed by ERIC-PCR. Logistic regression (univariate and multivariate) analyses were carried out to identify risk factors for human ESBL-PE carriage using a questionnaire survey amongst abattoir workers. ESBL-PE prevalence in animal samples from Cameroon were higher than for South Africa and ESBL-PE carriage was observed in Cameroonian workers only. Nasal ESBL-PE colonization was statistically significantly associated with hand ESBL-PE (21.95% vs. 91.67%; *p* = 0.000; OR = 39.11; 95% CI 2.02–755.72; *p* = 0.015). Low level of education, lesser monthly income, previous hospitalization, recent antibiotic use, inadequate handwashing, lack of training and contact with poultry were the risk factors identified. The study highlights the threat posed by ESBL-PE in the food chain and recommends the implementation of effective strategies for antibiotic resistance containment in both countries.

## 1. Introduction

*Enterobacteriaceae* are rod-shaped, Gram-negative bacteria, fermenting glucose, usually motile and facultative anaerobes, with the majority of genera being natural residents of gastrointestinal tract of animals, humans and some of them can be found in the environment [1,2]. The extensive use of third and fourth generation cephalosporins in human and animal health, has led to the emergence of extended spectrum β-lactamase-producing *Enterobacteriaceae* (ESBL-PE). ESBL-PE represent a significant public health concern globally and have recently been classified by the World Health Organization as pathogens of critical priority in research [3].

Several studies have detected ESBL-PE in food animals, especially pigs, poultry and cattle and food products throughout the world and their transmission from livestock to humans in the farm-to-plate continuum has been evidenced [4,5]. However, the prevalence of ESBL-PE in food animals and humans across the farm-to-plate continuum is yet to be elucidated in Sub-Saharan countries including Cameroon and South Africa. It is therefore imperative to understand the epidemiology and determine the burden of ESBL-PE in food animals in order to highlight the threat posed by these resistant bacteria and provide evidence for decision-makers to implement effective prevention and containment measures of antibiotic resistance (ABR) in Cameroon and South Africa. The objectives of this study were thus to assess and compare the colonization, antibiotic resistance profiles and genetic relatedness of ESBL-PE among pigs and exposed workers and delineate risk factors of ESBL-PE carriage in humans in these countries.

## 2. Results

### 2.1. Demographic Characteristics

Altogether, 114 people were contacted in the five selected slaughterhouses and 83 (73%) workers agreed to participate in the study, with the response rate being higher in Cameroon (71%) than in South Africa (59%). Table 1 describes nasal and hand ESBL-PE carriage of workers in relation to individual, medical/clinical history and slaughterhouse-related characteristics.

### 2.2. ESBL-PE Status in Humans

Out of the 53 workers sampled in Cameroon, 42 (79%) and 36 (68%) were colonized by hand and nasal ESBL-PE, respectively. The main species identified were *E. coli*, *Enterobacter spp.* and *K. pneumoniae* (Supplementary Table S1). In contrast, in South Africa, *Enterobacteriaceae* was not isolated from slaughterhouse workers.

Cameroonian isolates exhibited elevated resistance to ampicillin, trimethoprim-sulfamethoxazole, cefuroxime, cefuroxime-axetil, cefotaxime, ceftazidime and amoxicillin-clavulanic acid (Table 2) with no resistance observed against imipenem, ertapenem, meropenem and tigecycline (Table 2). The profiles AMP.TMP/SXT.CXM.CXM-A.CTX (34%) and AMP.AMC.TZP.CXM.CXM-A.CTX.CAZ.TMP/SXT (7%) were predominant in hand and nasal ESBL-*E. coli*, respectively, in humans in Cameroon (Table 3).

### 2.3. Epidemiological Background of ESBL-PE in Pigs

Overall, the prevalence of ESBL-PE in the pooled nasal and rectal samples was 75% (108/144) and 71% (102/144), respectively (Table 4). At country-level, 42% (30/72) and 50% (36/72) ESBL-PE were detected in rectal and nasal pooled samples in South Africa respectively, whereas a 100% ESBL-PE prevalence was isolated in both specimen types in Cameroon (Table 1). In Cameroon, the main species identified were *E. coli* (61%) and *Klebsiella pneumoniae* (25%) whereas in South Africa, *E. coli* was the sole *Enterobacteriaceae* species isolated in both types of samples (Supplementary Table S2).

ESBL-PE isolated from pigs exhibited high resistance to ampicillin, cefuroxime, cefuroxime-acetyl, cefotaxime, ceftazidime and trimethoprim-sulfamethoxazole in both countries (Table 2). One South African isolate expressed high resistance to colistin (8 mg/L) and no resistance to ertapenem, meropenem, imipenem and tigecycline was observed. The majority of ESBL-producing *E. coli* isolated from pigs in both countries showed the resistance profile AMP.TMP/SXT.CXM.CXM-A.CTX in both type of samples (Table 5).

### 2.4. Genotypic Relatedness

**Figure 1:**
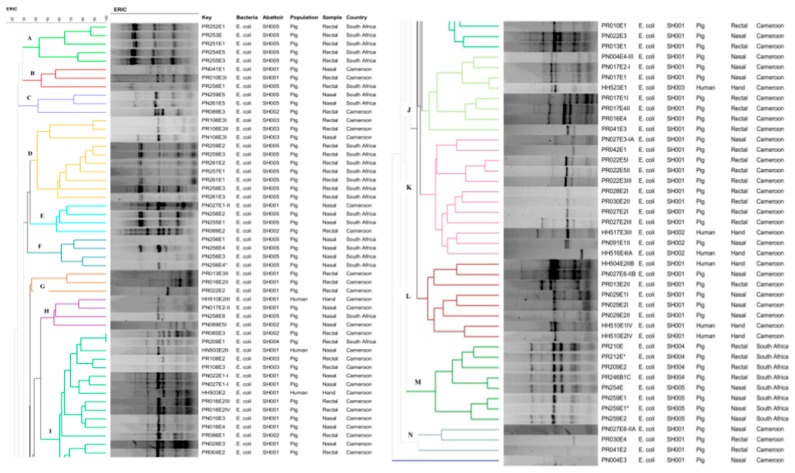
Genotypic relationship of ESBL-*E. coli* strains (n = 93) detected from exposed workers and pigs in Cameroon and South Africa. Dendogram generated by Bionumerics using UPGMA method and the Dice similarity coefficient.

ERIC-PCR allowed the differentiation of the 93 *E. coli* into 14 clusters named alphabetically from A-N (Figure 1). A batch of isolates in cluster M (PR210, PR212E *, PR209E2, PR246B1C and PN254E), collected from pigs of abattoir SH004 and SH005 in South Africa was considered to be closely related. Moreover, great interest was observed in cluster I, where one pair of animal strains, PR085E3 and PR209E1 isolated in abattoirs SH002 and SH004 in Cameroon and South Africa, respectively, showed 100% similarity and were closely related with a human strain (HN503E2II) detected in abattoir SH001 in Cameroon (Figure 1).

### 2.5. Risk Factors of Human ESBL-PE Carriage

Table 6 shows the relationship between ESBL-PE carriage in workers and the foremost putative risk factors. Nasal and hand ESBL-PE colonization were univariately associated with an odds ratio (OR) of 39.11 (95% CI 2.02–755.72; *p* = 0.015). Other determinants, univariately associated with nasal and hand ESBL-PE carriage were previous hospitalization, recent antibiotic use, inadequate handwashing, occupation of relatives and year in the employment. The multivariate analysis reveals that nasal and hand ESBL-PE carriage in humans were associated with contact with other animals, particularly poultry with high statistical significance for both sample types (OR = 5.83, 95% CI 1.58–21.48, *p* = 0.008; vs. OR = 8.41, 95% CI 2.27–31.11, *p* = 0.001).

## 3. Discussion

*Enterobacteriaceae* and especially ESBL-PE, were acknowledged as critical priority antibiotic-resistant bacteria (ARB) by the WHO and their emergence at the animal-human-environment interface presents a to serious and multifaceted public health concern globally [3]. This study investigated the carriage, risk factors, antibiotic resistance profiles and genetic relatedness of ESBL-PE isolated from apparently healthy pigs and occupationally exposed workers in Cameroon and South Africa.

The overall prevalence of human ESBL-PE carriage was 50% in hand and 45.75% in nasal samples. Comparable data was reported by Magoue et al. (2013) in Cameroon, where the prevalence of ESBL-PE faecal carriage was 45% in outpatients in the region of Adamaoua [6]. Our findings are nevertheless higher than that described by Dohmen et al. (2015) where a 27% prevalence of ESBL-PE carriage in faecal samples of people with daily exposure to pigs in Netherlands was described [7]. 

Our results are in contrast to a study of Fisher et al., (2016), where none of the 66.7% *Enterobacteriaceae* detected in the nares of participants were ESBL producers and where the authors concluded that nares were a negligible reservoir for colonization of ESBL-PE in pig’s exposed workers [8]. Our finding shows that the prevalence of ESBL-PE carriage in nasal samples substantially increased (8.33 vs. 91.67%; *p* < 0.001) and was statistically significantly correlated with their carriage on hand (OR 39.11; 95% CI 2.02–755.72; *p* = 0.015). In addition, nasal ESBL-PE carriage was associated with inappropriate handwashing with high statistical significance (OR 4.71; 95% CI 2.28–9.70; *p* < 0.001). This suggests, that nares might likely become reservoir of ESBL-PE when limited hygienic conditions prevail and biosecurity measures are not adequately implemented. It further reveals that, as with the transmission of nosocomial infections in hospital settings, hands constitute important vectors of ABR transmission in the food production industry and may not only drive the transfer from person-to-person but also the contamination of food products intended for the end consumer. Nasal ESBL-PE carriage reported herein might also be ascribed to airborne contamination as recently reported by Dohmen et al., (2017) who revealed that human CTX-M-gr1 carriage was statistically associated with presence of CTX-M-gr1 in dust (OR = 3.5, 95% CI = 0.6–20.9) and that inhalation of air might constitute another transmission route of ESBL-PE in the food chain [9]. 

The difference in the prevalence of ESBL-PE carriage in humans in both countries could be explained by the fact that South Africa has existing abattoir regulations in place and South African abattoirs were compliant with international food safety standard ISO 22000 and Hazard Analysis Critical Control Points (HACCP) plans. In Cameroon, slaughterhouse/markets were principally low-grade, lacking in basic amenities, with sub-optimal sanitary conditions and limited or non-existent biosecurity measures. The Food and Agriculture Organization for the United Nations (FAO) report on abattoir facilities in Central African countries including Cameroon, already underlined the gaps in term of biosecurity measures in these settings [10]. Our findings, therefore, reinforce the importance of and the need to implement strict biosecurity procedures as when effective prevention and containment measures are implemented, the risk of ABR dissemination is reduced.

The overall prevalence of ESBL-PE in pigs was 71% and 75% in rectal and nasal pooled samples, respectively. The results are consistent with that reported by Le et al., (2015) in food animals and products in Vietnam where a 68.4% prevalence of ESBL-producing *E. coli* was described [11]. They are however lower than that reported in pig farms in Germany, where 88.2% of ESBL-producing *E. coli* was detected [12] and higher than that reported in two other studies with prevalence ranging from 8.6 to 63.4% in food animals and food products in Netherlands, [13] and 8.4% in cattle in Switzerland [2]. 

The high rate of ESBL-PE carriage detected in both nasal and rectal samples in Cameroon may suggest that ESBL-PE are consistently widespread in food animals in Cameroon, disseminate in the farm-to-plate continuum and represent a grave public health threat in the country. Similarly, the ESBL-PE prevalence detected in pigs in South Africa is not surprising, especially because the use of antibiotics as growth promoter agents is legally allowed in the country [14]. These findings reveal gaps in the current state of knowledge about antibiotic use and ABR in food animals and suggests that the debate about ABR-related consequences in the farm-to-plate continuum is neglected in Cameroon and South Africa and should be more seriously considered in these countries. Additionally, our study revealed a high frequency (95%) of ESBL-producing *E. coli*, emphasizing the relevance of this indicator bacteria as a serious public health issue. 

ERIC analysis demonstrated relative associations amongst human and animal isolates within and across countries. Some strains isolated in humans were highly related to those detected from pigs at similar or dissimilar abattoirs suggesting that the occurrence of ESBL-PE in humans may have an animal origin or vice-versa and that these bacteria may spread to humans via the food chain, allowing their dissemination to the global population. Although not providing evidence on the transmission dynamics of ESBL-PE, our results nevertheless show an epidemiological link amongst isolates from humans and animals. 

Hospitalization, antibiotic use and contact with (food) animals are known risk factors for human ESBL-PE carriage [15]. Twenty-one abattoir workers or their family members had been admitted to a hospital within the year of the sampling. Of these, 39.29% evidenced nasal ESBL-PE carriage and 71.43% hand ESBL-PE colonization (Table 1). Likewise, the majority of workers who had used antibiotics within the month of the sampling were colonized by ESBL-PE in nares (55.26%) and hands (71.05%) (Table 1). 

There are certain limitations to consider in this cross-sectional study. First, the duration of ESBL-PE carriage was not investigated and there was no apparent relationship between human ESBL-PE carriage and contact with ESBL-PE colonized pigs (Table 6). Secondly, in contrast, a clear association was established between contact with other (food) animals, mainly poultry and human ESBL-PE colonization, with high statistical significance (Table 6), suggesting that further work should be undertaken in high risk populations and other food animals such as poultry in order to expand our understanding on the public health impact of the likely zoonotic transmission of ESBL-PE through the farm-to-plate continuum. Thirdly, the small human sample size precluded any direct conclusions on the prevalence of antibiotic resistance among abattoir workers. Finally, the molecular analyses were only carried out on a representative sub-sample and not all isolates due to financial constraints. Comprehensive molecular analysis would have certainly allowed better understanding of the genetic exchanges and evolution that are likely to occur within and between bacteria in this continuum.

To the best of our knowledge, this is the first report of ESBL-PE in animals and humans in both Cameroon and South Africa taking food safety perspective. The high prevalence of ESBL-PE found in pigs in both countries as well as in humans in Cameroon highlights the food safety issue associated with their presence in the farm-to-plate continuum. It demonstrates the urgent need to implement multi-sectorial, multi-faceted and sustainable collaboration and activities among all stakeholders involved in this continuum in order to reduce the prevalence and contain the dissemination of ESBL-PE and ABR in these countries.

## 4. Methods

### 4.1. Study Design and Study Sites

From March to October 2016, a multicentre study was conducted in five abattoirs in Cameroon (n = 3) and South Africa (n = 2). All abattoirs were coded for ethical reasons as SH001, SH002, SH003, SH004 and SH005. They were visited thrice at different time points to allow a representative sample. 

### 4.2. Ethical Considerations

Prior to the implementation of the study, ethical approvals were obtained from the National Ethics Committee for Research in Human Health of Cameroon (Ref. 2016/01/684/CE/CNERSH/SP), Biomedical Research Ethics Committee (Ref. BE365/15) and Animal Research Ethics Committee (Ref. AREC/091/015D) of the University of KwaZulu-Natal. In addition, ministerial approvals from the Cameroonian Ministry of Scientific Research and Innovation (Ref. 015/MINRESI/B00/C00/C10/C14) and Ministry of Livestock, Fisheries and Animal Industries (Ref. 061/L/MINEPIA/SG/DREPIA/CE) were also granted. This study was further placed on record with the South African National Department of Agriculture, Forestry and Fisheries [Reference: 12/11/1/5 (878)].

### 4.3. Sampling Procedures and Survey

#### 4.3.1. Animal Sampling Procedure

Apparently healthy and freshly slaughtered/stunned pigs were randomly sampled in both Cameroon and South Africa. The interior cavity of both anterior nares were swabbed and rectal swabs of pigs were obtained using sterile Amies swabs (Copan Italia Spa, Brescia, Italia). Altogether, 432 nasal and rectal pigs were collected in both countries, with the number of samples from each slaughterhouse (SH001, n = 129; SH002, n = 57; SH003, n = 30; SH004, n = 120; SH005, n = 96) proportional to the annual pig production per site.

#### 4.3.2. Human Sampling Procedure

Total sampling was employed where all exposed workers (≥21 years old) willing to participate were recruited in the study upon oral and written informed consent. Participants were requested to answer a questionnaire describing socio-demographic and medical/clinical history, as well as probable risk factors associated with ESBL-PE emergence/colonization and spread. Amies swab was used to collect both anterior nares and hand (between fingers for each right and left hand) samples which were processed within 4 h of collection.

### 4.4. Bacteriological Analysis

For the bacteriological analysis, three individual pig samples were pooled per abattoir, gender, specimen and area of breeding leading to 288 pools (144 nasal and 144 rectal) representing 432 original specimens collected from 432 pigs. Pooled pig samples and human swabs were cultured onto an in-house selective MacConkey agar supplemented with 2 mg/L cefotaxime (MCA+CTX) and incubated for 18–24 h at 37 °C for ESBL-PE screening. Presumptive ESBL-PE were phenotypically confirmed with Vitek^®^ 2 System (BioMérieux, Marcy l’Etoile, France). 

### 4.5. ESBL Detection, Species Identification and Antimicrobial Susceptibility Testing

Each colony with a unique morphotype growing on MCA+CTX was screened for ESBL production through the standard double disk synergy test (DDST) as recommended by the Clinical Laboratory and Standards Institute (CLSI) [16].

A panel of 19 antibiotics including amoxicillin + clavulanic acid, ampicillin, cefuroxime, cefuroxime axetil, cefoxitin, cefotaxime, ceftazidime, cefepime, imipenem, ertapenem, meropenem, amikacin, gentamicin, ciprofloxacin, tigecycline, piperacillin/tazobactam, nitrofurantoin, colistin and trimethoprim-sulfamethoxazole, were tested using Vitek^®^ 2 System and Vitek^®^ 2 Gram Negative Susceptibility card (AST-N255) (BioMérieux, Marcy l’Etoile, France). The CLSI was used for interpretation of the results excepted for colistin, piperacillin/tazobactam, amoxicillin + clavulanic acid and amikacin that were interpreted using EUCAST breakpoints [17]. *E. coli* ATCC 25922 was used as the control. 

### 4.6. Genotypic Relatedness Determination of ESBL-Producing Escherichia coli

The Thermo Scientific^®^ GeneJet Genomic DNA purification kit (Thermo Fisher Scientific, Johannesburg, South Africa) was used for the genomic DNA extraction following the manufacturer’s instructions. ERIC-PCR was carried out using the primers ERIC 1 5′-ATG TAA GCT CCT GGG GAT TCA C-3′ and ERIC 2 5′-AAG TAA GTG ACT GGG GTG AGC G-3′ [18]. Reactions were performed in a 10 µL final solution containing 5 µL Dream*Taq* Green Polymerase Master Mix 2× (Thermo Fisher Scientific, South Africa), 2.8 µL nuclease free water, 0.1 µL of each primer (100 μM) and 2 µL DNA template and run in an Applied Biosystems 2720 programmable thermal cycler (Thermo Fisher Scientific, Johannesburg, South Africa). The ERIC-PCR protocol implemented included 3 min of initial denaturation at 94 °C, followed by 30 cycles consisting of a denaturation at 94 °C for 30 s, annealing at 50 °C for 1 min, extension at 65 °C for 8 min, a final extension at 65 °C for 16 min and final storage at 4 °C. ERIC profiles were digitized and analysed using Bionumerics (version 7.6, Applied Maths, Austin, TX, USA). The similarity between each strain was assessed using Dice coefficient and dendrograms were constructed using the Unweighted Pair-Group Method Algorithm (UPGMA).

### 4.7. Data Analysis

Data was encoded and entered into Epi Info (version 7.2, CDC, Atlanta, GA, USA) and Excel (Microsoft Office 2016) and analysed using STATA (version 14.0, STATACorp LLC, College Statioon, TX, USA). A data set was designed for specific human results and, combined animal and abattoir data. Abattoirs were classified as ESBL-positive if an ESBL-PE was identified from at least one pool (nasal or rectal samples). Likewise, each human was categorized as carrier or non-carrier, with carrier being defined as having ESBL-PE in at least one site (nares or hand).

The ESBL-PE prevalence was compared between categories with the chi square test (*p* < 0.05). The relationship between ESBL-PE carriage in pigs and humans was ascertained using logistic regression analyses adjusted for clustering at abattoir level. Likewise, risk factors for ESBL-PE carriage were determined univariately and selected for multivariate analysis when the *p*-value was <0.2. The McFadden’s pseudo R^2^ statistic (maximum likelihood method) was used to check the model fit and the final model included all determinants for which the pseudo R^2^ was the most elevated with *p* < 0.05 for each dependent variable.

## Figures and Tables

**Table 1:** Nasal and hand ESBL-PE carriage of workers in relation to personal, medical/clinical and slaughterhouse-related characteristics. Of 84 workers enrolled, one withdrew prior to the sample collection and six refused the nasal sampling, yielding a total of 83 hand and 77 nasal samples. A few workers could not recall precise information whilst other refused to answer some questions leading to missing information that was not considered in the analysis.

Variables	Nasal Sample			Hand Sample		
	Frequency n (%)	Prevalence ESBL-PE (%)	Overall *p*-Value	Frequency n (%)	Prevalence ESBL-PE (%)	Overall *p*-Value
**Personal characteristics**						
***Country***						
Cameroon	53 (69)	67.92	**0.000**	53 (64)	79	**0.000**
South Africa	24 (31)	0		30 (36)	0	
***Gender***						
Female	9 (12)	44.44	0.883	12 (12)	41.67	0.503
Male	68 (88)	47.06		71 (88)	52.11	
***Age***						
21–30	31 (40)	41.94	0.084	32 (39)	46.88	0.063
31–40	26 (34)	50		28 (34)	42.86	
41–50	13 (17)	38.46		14 (17)	71.43	
51–60	5 (6)	100		6 (7)	83.33	
Above 60	2 (3)	0		3 (3)	0	
***Educational level***						
Illiterate	4 (5)	50	**0.048**	5 (6)	40	**0.032**
Primary school not achieved	6 (8)	50		7 (8)	42.86	
Primary school	34 (44)	64.71		35 (42)	71.43	
Secondary school	27 (35)	25.93		27 (33)	37.04	
High school/university	6 (8)	33.33		8 (10)	25	
***Average monthly income (US $)***						
Below 55	8 (10)	62.50	**0.007**	8 (14)	62.5	**0.004**
55–110	14 (19)	78.57		14 (29)	85.71	
110–165	12 (16)	66.67		12 (17)	58.33	
165–220	10 (13)	40		10 (17)	70	
220–275	20 (27)	20		24 (10)	25	
Above 275	11 (15)	27.27		13 (12)	30.77	
***Relative working at hospital or with animals***						
Yes	42 (55)	64.29	**0.001**	44 (53)	68.18	**0.001**
No	35 (45)	25.71		39 (47)	30.77	
**Clinical factors**						
***Recent hospitalization (within one year of sampling)***						
Yes	21 (27)	39.29	**0.032**	21 (25)	71.43	**0.027**
No	56 (73)	66.67		62 (75)	43.55	
***Nasal problem***						
Yes	11 (14)	36.36	0.456	11 (13)	45.45	0.714
No	66 (86)	48.48		72 (87)	51.39	
***Skin problem***						
Yes	14 (18)	28.57	0.132	14 (17)	35.71	0.222
No	63 (82)	50.76		69 (83)	53.62	
***Recent antibiotic use (month prior the sampling)***						
Yes	38 (49)	55.26	0.140	38 (64)	71.05	**0.001**
No	39 (51)	38.46		45 (36)	33.33	
**Slaughterhouse-related factors**						
***Closeness of abattoir with house***						
Yes	32 (42)	40.63	0.363	14 (33)	17	0.184
No	45 (58)	51.11		28 (67)	34	
***Abattoir***						
SH001	21 (27)	76.19	**0.000**	21 (25)	85.71	**0.000**
SH002	19 (25)	36.84		19 (23)	63.16	
SH003	13 (17)	100		13 (16)	92.31	
SH004	4 (5)	0		10 (12)	0	
SH005	20 (26)	0		20 (24)	0	
***Principal activity or working area***						
Slaughterer	34 (44)	58.82	**0.012**	34 (41)	58.82	**0.000**
Transport of pig/pork	5 (7)	80		5 (6)	80	
Wholesaler	7 (9)	28.57		7 (8)	85.71	
Butcher	5 (7)	80		5 (6)	80	
Retailer of viscera *	7 (9)	71.43		7 (8)	85.71	
Retailer of grilled pork ^#^	1 (1)	0		1 (1)	100	
Scalding of pigs	3 (4)	0		3 (4)	0	
Evisceration	8 (10)	0		14 (17)	0	
Transport of viscera/blood	1 (1)	0		1 (1)	0	
Veterinarian	5 (7)	20		5 (6)	20	
Meat inspector	1 (1)	0		1 (1)	0	
***Training to practice profession***						
Yes	28 (36)	3.57	**0.000**	34 (41)	2.94	**0.000**
No	49 (64)	71.43		49 (59)	83.67	
***Year in profession***						
[0–4]	31 (43)	35.48	0.356	31 (39)	38.71	0.357
[5–9]	6 (8)	66.67		8 (10)	50	
[10–14]	22 (30)	50		24 (30)	58.33	
Above 15	14 (19)	57.14		16 (20)	62.50	
***Intensity of pig’s contact (rare, low, frequent, very frequent)***						
Always	35 (45)	51.43	0.348	35 (42)	57.14	0.136
Almost always	32 (42)	37.50		38 (46)	39.47	
Sometimes	10 (13)	60		10 (12)	70	
***Contact with other animals during handling or various procedures of processing of animals at the abattoir***						
Yes	38 (50)	60.53	**0.046**	39 (48)	69.23	**0.004**
No	38 (50)	34.21		42 (52)	35.71	
***Intensity of contact with other animals***						
Always	8 (21)	87.50	**0.025**	8 (20)	100	**0.006**
Almost always	9 (24)	22.22		10 (26)	30	
Sometimes	17 (45)	58.82		17 (44)	70.59	
Rarely	4 (10)	100		4 (10)	100	

* retailer of viscera: street-vendor buying pig’s viscera from abattoir workers, performing manual cleaning in order to sells ready-to-eat meal; **^#^* retailer of grilled pork*:** street-vendor acquiring pork at the slaughterhouse in order to sells ready-to-eat grilled pork.

**Table 2:** Antimicrobial susceptibility results of extended-spectrum β-lactamase-producing *Enterobacteriaceae* (ESBL-PE) isolated from pigs and humans.

Antibiotics	Cameroon				South Africa	
	Pig		Human		Pig	
	MIC (µg/mL) Range	No. (%) Resistant Isolates	MIC (µg/mL) Range	No. (%) Resistant Isolates	MIC (µg/mL) Range	No. (%) Resistant Isolates
Ampicillin	≥32	126 (95)	≤2–≥32	32(73)	≥32	38 (100)
Amoxicillin-clavulanate	4–≥32	54(40)	≤2–≥32	8(18)	8–16	2(5)
Piperacillin-tazobactam	≤4–≥128	24(18)	≤4–64	2(5)	≤4	0
Cefuroxime	4–≥64	124(93)	≤1–≥64	19(43)	≥64	38 (100)
Cefuroxime-axetil	4–≥64	125(93)	≤1–≥64	19(43)	≥64	38 (100)
Cefoxitin	≤4–≥64	10(7)	≤4–≥64	3(7)	≤4	0
Cefotaxime	≤1–≥64	118(88)	≤1–≥64	14(32)	4–≥64	38 (100)
Ceftazidime	≤1–≥64	93(69)	≤1–≥64	8(18)	≤1–4	1 (3)
Cefepime	≤0.5–≥64	6(4)	≤1–≥64	2(5)	≤1–4	1 (3)
Meropenem	≤0.25	0	≤0.25	0	≤0.25	0
Imipenem	≤0.25	0	≤0.25	0	≤0.25	0
Ertapenem	≤0.5	0	≤0.5	0	≤0.5	0
Amikacin	≤2–16	11(8)	≤2–16	1(2)	≤2–16	1 (3)
Gentamicin	≤1–≥16	43(32)	≤1–≥16	3(7)	≤1–≥16	7(18)
Ciprofloxacin	≤0.25–≥4	33(25)	≤0.25–≥4	2(5)	≤0.25	0
Tigecycline	≤0.5–2	0	≤0.5–1	0	≤0.5–1	0
Nitrofurantoin	≤16–64	0	≤16–128	1(2)	≤16–64	0
Colistin	≤0.5	0	≤0.5–4	1(2)	≤0.5–8	1(3)
Trimethoprim-sulfamethoxazole	≤20–≥320	119 (89)	≤20–≥320	22(50)	≤20–≥320	36(95)

**Table 3:** Antimicrobial resistance profiles of extended-spectrum β-lactamase-producing *Enterobacteriaceae* (ESBL-PE) strains isolated from humans.

Bacteria	Resistance Profiles	Cameroon	
		Nasal, n (%)	Hand, n (%)
***E. coli***	AMP.AMC.TZP.CXM.CXM-A.CTX.CAZ.TMP/SXT	1 (50)	0
	AMP.CXM.CXM-A.CAZ.CS	0	1(8)
	AMP.TMP/SXT.CXM.CXM-A.CTX	0	4(31)
	AMP.TMP/SXT.CXM.CXM-A.CTX.CAZ.AMC.GM.CIP.FEP	0	1(8)
	AMP.TMP/SXT.CXM.CXM-A.CTX.CAZ.AMC.TZP	0	1(8)
	AMP.TMP/SXT.CXM.CXM-A.CTX	0	1(8)
	AMP.CXM.CXM-A.CTX.FEP.TMP/SXT	0	1(8)
***E. dissolvens***	AMP.AMC.CXM.CXM-A.FOX.CTX.TMP/SXT	1 (50)	0
***S. sonnei***	AMP.TMP/SXT.CXM.CXM-A.CTX.CAZ	0	1(8)
***K. pneumoniae***	AMP.AMC.CXM.CXM-A.CTX.TMP/SXT.GM.FT	0	1(8)
	AMP.AMC.CXM.CXM-A.CTX.CAZ.AN.GM.CIP	0	1(8)
	AMP.TMP/SXT.CXM.CXM-A.CTX.CAZ	0	1(8)
**Grand Total**		2 (100)	13 (100)

AMP: Ampicillin; AMC: Amoxicillin-clavulanate; TZP: Piperacillin-tazobactam; CXM: Cefuroxime; CXM-A: Cefuroxime-Acetyl; CTX: Cefotaxime; CAZ: Ceftazidime; TMP/SXT: Trimethoprim-Sulfamethoxazole; FOX: Cefoxitin; GN: Gentamicin; CIP: Ciprofloxacin; FEP: Cefepime; CS: Colistin.

**Table 4:** Extended-spectrum β-lactamase-producing *Enterobacteriaceae* (ESBL-PE) in pooled nasal and rectal samples.

Characteristics	Nasal Samples			Rectal Samples		
	Frequency Pooled Samples, n (%)	Nasal ESBL, n (%)	Overall *p*-Value	Frequency Pooled Samples, n (%)	Rectal ESBL, n (%)	Overall *p*-Value
**Country**						
Cameroon	72 (50)	72 (100)	**0.000**	72 (50)	72 (100)	**0.000**
South Africa	72 (50)	36 (50)		72 (50)	30 (41.67)	
**Abattoir**						
SH001	43 (30)	43 (100)	**0.000**	43 (30)	43 (100)	**0.000**
SH002	19 (13)	19 (100)		19 (13)	19 (100)	
SH003	10 (7)	10 (100)		10 (7)	10 (100)	
SH004	40 (28)	19 (47.50)		40 (28)	9 (22.50)	
SH005	32 (22)	17 (53.13)		32 (22)	21 (65.63)	
**Gender**						
Sow	79 (55)	64 (81.01)	0.066	79 (55)	59 (74.68)	0.262
Boar	65 (45)	44 (67.69)		65 (45)	43 (66.15)	
**Time point**						
First	42 (29)	31 (73.81)	0.149	42 (29)	34 (80.95)	**0.050**
Second	54 (38)	45 (83.33)		54 (38)	40 (74.07)	
Third	48 (33)	32 (66.67)		48 (33)	28 (58.33)	

**Table 5:** Antimicrobial resistance profiles of extended-spectrum β-lactamase-producing *Enterobacteriaceae* (ESBL-PE) detected from pigs.

Bacteria	Resistance Profiles	No. Antibiotics	No. Classes	Cameroon		South Africa	
				Nasal, n (%)	Rectal, n (%)	Nasal, n (%)	Rectal, n (%)
***E. coli***	AMP.TMP/SXT.CXM.CXM-A.CTX	5	2	3 (5)	2 (4)	0	29(94)
	AMP.TMP/SXT.CXM.CXM-A.CTX.CAZ	6	2	7 (12)	11 (20)	0	0
	AMP.TMP/SXT.CXM.CXM-A.CTX.CAZ.FEP	7	2	2 (3)	2 (4)	0	0
	AMP.TMP/SXT.CXM.CXM-A.CTX.CAZ.GM.CIP	8	4	1 (2)	2 (4)	0	0
	AMP.TMP/SXT.CXM.CXM-A.CTX.CAZ.CIP	7	3	1 (2)	0	0	0
	AMP.TMP/SXT.CXM.CXM-A.CTX.CAZ.AMC	8	3	1 (2)	3 (5)	0	0
	AMP.TMP/SXT.CXM.CXM-A.CTX.CAZ.AMC.GM.CIP	9	4	2 (3)	3 (5)	0	0
	AMP.TMP/SXT.CXM.CXM-A.CTX.CAZ.AMC.TZP	8	2	5 (8)	5 (9)	0	0
	AMP.TMP/SXT.CXM.CXM-A.CTX.CAZ.AMC.TZP.FOX.FEP.GM.CIP	12	4	1 (2)	0	0	0
	AMP.CXM.CXM-A.CTX.CAZ	5	1	0	2 (4)	0	0
	AMP.TMP/SXT.CXM.CXM-A.CTX.CAZ.FEP.GM.	8	3	0	1 (2)	0	0
	AMP.TMP/SXT.CXM.CXM-A.CTX.CIP	6	3	0	1 (2)	0	0
	AMP.TMP/SXT.CXM.CXM-A.CTX.CAZ.CIP.AMC	8	3	0	5 (9)	0	0
	AMP.TMP/SXT.CXM.CXM-A.CTX.GM	6	3	0	1 (2)	0	0
	AMP.TMP/SXT.CXM.CXM-A.CTX.CAZ.CIP.AMC.TZP	9	3	0	1 (2)	0	0
	AMP.TMP/SXT.CXM.CXM-A.CTX.CAZ.CIP.GM.TZP.FEP	10	4	0	1 (2)	0	0
	AMP.CXM.CXM-A.CTX	4	1	0	0	0	2 (6)
	AMP.CXM.CXM-A.CTX.TMP/SXT.CAZ.FEP.AK.GM.CS	10	4	0	0	1 (14)	0
	AMP.CXM.CXM-A.CTX.TMP/SXT.GM	6	3	0	0	1 (14)	0
	AMP.CXM.CXM-A.CTX.TMP/SXT.GM.AMC	7	3	0	0	5 (71)	0
	AMP.TMP/SXT.CXM.CXM-A.CTX.CAZ.CIP.GM.AMC.TZP	10	4	0	2 (4)	0	0
***K. pneumoniae***	AMP.TMP/SXT.CXM.CXM-A.CTX.GM	6	3	2 (3)	1 (2)	0	0
	AMP.TMP/SXT.CXM.CXM-A.CTX.CAZ.AK.GM.CIP.AMC	10	4	0	6 (11)	0	0
	AMP.TMP/SXT.CXM.CXM-A.CAZ.AMC.TZP	7	2	0	1 (2)	0	0
	AMP.TMP/SXT.CXM.CXM-A.CTX.CAZ.GM.AMC.TZP	9	3	4 (7)	2 (4)	0	0
	AMP.TMP/SXT.CXM.CXM-A.CTX.CAZ.AMC.TZP.AK.GM.CIP	11	4	0	3(5)	0	0
	AMP.TMP/SXT.CXM.CXM-A.CTX	5	2	1 (2)	0	0	0
	AMP.TMP/SXT.CXM.CXM-A.CTX.CAZ	6	2	4 (7)	0	0	0
	AMP.TMP/SXT.CXM.CXM-A.CTX.CAZ.GM	7	3	8 (14)	0	0	0
	AMP.TMP/SXT.CXM.CXM-A.CTX.CAZ.GM.AMC.TZP	9	3	4 (7)	0	0	0
***K. ozaenae***	AMP.TMP/SXT.CXM.CXM-A.CTX.CAZ.AK.GM.CIP.AMC	10	4	1 (2)	0	0	0
***Enterobacter cloacae***	AMP.AMC.CXM.CXM-A.FOX.CTX.TMP/SXT	7	2	4 (7)	0	0	0
***C. freundii***	AMP.AMC.CXM.CXM-A.FOX.CTX.TMP/SXT	7	2	3 (5)	0	0	0
***S. sonnei***	AMP.CTX.CAZ.TMP/SXT	4	2	1 (2)	0	0	0
	AMP.TMP/SXT.CXM.CXM-A.CTX.CAZ	6	2	4 (7)	0	0	0

AMP: Ampicillin; AMC: Amoxicillin-clavulanate; AK: Amikacin; CXM: Cefuroxime; CXM-A: Cefuroxime-acetyl; CTX: Cefotaxime; CAZ: Ceftazidime; CS: Colistin; CIP: Ciprofloxacin; FEP: Cefepime; FOX: Cefoxitin; GM: Gentamicin; TMP/SXT: Trimethoprim-sulfamethoxazole; TZP: Piperacillin-tazobactam.

**Table 6:** Predictors of nasal and hand ESBL-PE carriage among humans. Univariate and multivariate analysis (logistic regression).

Variables	Univariate Logistic Regression Analysis				Multivariate Logistic Regression Analysis			
	Nasal ESBL-PE Carriage		Hand ESBL-PE Carriage		Nasal ESBL-PE Carriage		Hand ESBL-PE Carriage	
	OR (95% CI)	*p*	OR (95% CI)	*p*	OR (95% CI)	*p*	OR (95% CI)	*p*
Abattoir	0.43 (0.22–0.85)	**0.014**	0.28 (0.15–0.55)	**0.000**	2.54 (0.47–17.75)	0.254	1.75 (0.19–15.54)	0.615
Gender	1.11 (0.10–12.39)	0.932	1.52 (0.20–11.38)	0.682	8.74 (1.03–74.16)	**0.047**	27.94 (1.68–463.05)	**0.020**
Educational level	0.61 (0.39–0.94)	**0.024**	0.72 (0.56–0.91)	**0.006**				
Monthly Income	0.57 (0.36–0.89)	**0.014**	0.60 (0.36–0.99)	**0.045**	0.58 (0.35–0.97)	**0.039**	0.76 (0.44–1.31)	0.324
Training	0.01 (0.0003–0.79)	**0.038**	0.006 (0.0005–0.0658)	**0.000**	0.004 (0.00009–0.22)	**0.006**	0.0008 (0.000008–0.09)	**0.003**
Principal Activities	0.63 (0.50–0.78)	**0.000**	0.63 (0.46–0.87)	**0.004**				
Occupation of relative ^a^	5.2 (1.46–18.56)	**0.011**	4.82 (0.64–36.56)	0.128	5.62 (1.02–30.82)	**0.047**	3.58 (0.57–22.43)	0.172
Year in Profession	1.40 (0.66–2.97)	0.387	1.35 (0.68–2.69)	0.398				
Age	1.09 (0.80–1.48)	0.595	1.07 (0.61–1.89)	0.817				
Recent hospitalization ^b^	3.09 (1.26–7.59)	**0.014**	3.24 (1.18–8.86)	**0.022**	1.28 (0.24–6.87)	0.769	0.57 (0.08–4.12)	0.576
Recent antibiotic use ^c^	1.97 (0.40–9.73)	0.402	4.91 (1.20–20.03)	**0.027**				
Skin problem	0.39 (0.17–0.89)	**0.025**	0.48 (0.21–1.08)	0.076				
Nasal problem	0.61 (0.29–1.28)	0.192	0.79 (0.34–1.82)	0.578				
Protective working clothes	0.04 (0.002–0.812)	**0.036**	0.022 (0.002–0.258)	**0.002**				
Inadequate Handwashing	4.71 (2.28–9.70)	**0.000**	3.9 (1.01–15.01)	**0.048**				
Convenient handwashing	0.08 (0.017–0.41)	**0.002**	0.04 (0.013–0.145)	**0.000**				
Intensity of contact with pigs	0.97 (0.50–1.87)	0.920	0.96 (0.40–2.31)	0.934				
Contact with other animals	2.95 (0.87–10.04)	0.084	4.05 (1.42–11.53)	**0.009**				
Contact with poultry	5.83 (1.58–21.48)	0.008	8.41 (2.27–31.11)	**0.001**	9.93 (1.37–71.63)	**0.023**	24.22 (1.28–457.35)	**0.034**
Pig colonization Nasal ESBL (yes or No)	1.04 (0.93–1.16)	0.509	1.06 (0.95–1.17)	0.313				
Pig colonization Rectal ESBL (yes or No)	1.03 (0.93–1.15)	0.585	1.05 (0.96–1.16)	0.273				

^a^: Relative working with food animals, food products or at hospital, ^b^: Within one year prior the sampling date; c: Within one month prior the sampling date.

## Supplementary Materials

The following are available online at http://www.mdpi.com/2076-0817/8/1/10/s1. Table S1: Overall prevalence of extended spectrum β-lactamase (ESBL) producing bacteria isolated from humans per country and specimen type. Table S2: Overall prevalence of extended spectrum β-lactamase (ESBL) producing bacteria isolated from animals per country and specimen type. Table S3: Prevalence and distribution of extended-spectrum β-lactamase producing-E. coli clusters per abattoir.

## References

[1] Aidara-Kane A., Andremont A., Collignon P. (2013). Antimicrobial resistance in the food chain and the AGISAR initiative. J. Infect. Public Health.

[2] Reist M., Geser N., Hächler H., Schärrer S., Stephan R. (2013). ESBL-producing *Enterobacteriaceae*: Occurrence, risk factors for fecal carriage and strain traits in the swiss slaughter cattle population younger than 2 years sampled at abattoir level. PLoS ONE.

[3] World Health Organization (WHO) (2017). Global Priority List of Antibiotic Resistant Bacteria to Guide Research, Discoveries and Development of New Antibiotics.

[4] Founou L.L., Founou R.C., Essack S.Y. (2016). Antibiotic resistance in the food chain: A developing country-perspective. Front. Microbiol..

[5] Ewers C., Bethe A., Semmler T., Guenther S., Wieler L.H. (2012). Extended-spectrum β-lactamase-producing and AmpC-producing *Escherichia coli* from livestock and companion animals, and their putative impact on public health: A global perspective. Clin. Microbiol. Infect..

[6] Magoué C.L., Melin P., Gangoué-Piéboji J., Okomo Assoumou M.C., Boreux R., De Mol P. (2013). Prevalence and spread of extended-spectrum β-lactamase-producing *Enterobacteriaceae* in Ngaoundere, Cameroon. Clin. Microbiol. Infect..

[7] Dohmen W., Bonten M.J., Bos M.E., van Marm S., Scharringa J., Wagenaar J.A., Heederik D.J. (2015). Carriage of extended-spectrum β-lactamases in pig farmers is associated with occurence in pigs. Clin. Microbiol. Infect..

[8] Fischer J., Hille K., Mellmann A., Schaumburg F., Kreienbrock L., Köck R. (2016). Low-level antimicrobial resistance of *Enterobacteriaceae* isolated from the nares of pig-exposed persons. Epidemiol. Infect..

[9] Dohmen W., Schmitt H., Bonten M., Heederik D. (2017). Air exposure as a possible route for ESBL in pig farmers. Environ. Res..

[10] Food and Agriculture Organization for the United Nations (FAO) (2013). Étude sur les Abattoirs D’animaux de Boucherie en Afrique Centrale (Cameroun–Congo–Gabon–Tchad).

[11] Le H.V., Kawahara R., Khong D.T., Tran H.T., Nguyen T.N., Pham K.N., Jinnai M., Kumeda Y., Nakayama T., Ueda S. (2015). Widespread dissemination of extended-spectrum β-lactamase-producing, multidrug-resistant *Escherichia coli* in livestock and fishery products in vietnam. Int. J. Food Contam.

[12] Dahms C., Hübner N.O., Kossow A., Mellmann A., Dittmann K., Kramer A. (2015). Occurrence of ESBL-producing *Escherichia coli* in livestock and farm workers in mecklenburg-Western Pomerania, Germany. PLoS ONE.

[13] Geser N., Stephan R., Hachler H. (2012). Occurrence and characteristics of extended-spectrum β-lactamase (ESBL) producing *Enterobacteriaceae* in food producing animals, minced meat and raw milk. BMC Vet. Res..

[14] Department of Agriculture, Forestry and Fisheries, South Africa (DAFF) (1996). Fertilizer, Farm Feeds, Agricultural Remedies and Stock Remedies Act, 1947.

[15] Ben-Ami R., Rodríguez-Baño J., Arslan H., Pitout J.D., Quentin C., Calbo E.S., Azap Ö.K., Arpin C., Pascual A., Livermore D.M. (2009). A multinational survey of risk factors for infection with extended-spectrum β-lactamase-producing *Enterobacteriaceae* in non-hospitalized patients. Clin. Infect. Dis..

[16] Clinical Laboratory Standards Institute (CLSI) (2016). Performance Standards for Antimicrobial Susceptibility Testing. Proceedings of the Twenty-Sixth Informational Supplement.

[17] European Committee on Antimicrobial Susceptibility Testing (EUCAST) (2016). Breakpoint Tables for Interpretation of MICs and zone Diameters Version 6.1. http://www.eucast.org/fileadmin/src/media/PDFs/EUCAST_files/Breakpoint_tables/v_6.0_Breakpoint_Tables.pdf.

[18] Versalovic J., Koeuth T., Lupski J.R. (1991). Distribution of repetitive DNA sequences in eubacteria and application to fingerprinting of bacterial genomes. Nucleic Acids Res..

