# Supplementary material for: Emergence and Spread of Extended Spectrum β-Lactamase Producing Enterobacteriaceae (ESBL-PE) in Pigs and Exposed Workers: A Multicentre Comparative Study between Cameroon and South Africa
Source: Pathogens. 2019 Jan 16;8(1):10. doi: 10.3390/pathogens8010010 (PMC6470761; doi:10.3390/pathogens8010010)
Supplement: Supplementary file 1 [file pathogens-08-00010-s001.pdf]

Table S1. Overall prevalence of extended spectrum β-lactamase (ESBL) producing bacteria isolated from humans per country and specimen type

**Bacteria**	**Cameroon**		**South Africa**	
	**Hand (%)**	**Nasal (%)**	**Hand (%)**	**Nasal (%)**
E. coli	16 (41)	15 (47)	0	0
E. dissolvens	2 (5)	1 (3)	0	0
K. pneumoniae	2 (5)	6 (19)	0	0
Shigella sonnei	1 (3)	1 (3)	0	0
Others	18 (46)	9 (28)	10 (50)	10 (50)
**Total**	39 (100)	32 (100)	10 (100)	10 (100)

Table S2. Overall prevalence of extended spectrum β-lactamase (ESBL) producing bacteria isolated from animals per country and specimen type

**Bacteria**	**Cameroon**		**South Africa**	
	**Nasal (%)**	**Rectal (%)**	**Nasal (%)**	**Rectal (%)**
Bordetella bronchoseptica	0	0	33 (60)	2 (5)
C. freundii	3 (3)	0	0	0
E. coli	29 (32)	42 (63)	10 (18)	17 (42.5)
Enterobacter cloacae dissolvens	8 (9)	0	0	0
K. ozaenae	1 (1)	0	0	0
K. pneumoniae	19 (21)	15 (22)	0	0
S. sonnei	1 (1)	4 (6)	0	0
Others	29 (32)	6 (9)	12 (22)	21 (52.5)
Total	91 (100)	67 (100)	55 (100)	40 (100)

Table S3. Prevalence and distribution of extended-spectrum β-lactamase producing-*E. coli* clusters per abattoir.

Cluster	Resistance profiles	No. isolates	No. isolates (%)	Abattoir				
				SH001	SH002	SH003	SH004	SH005
A	AMP.TMP/SXT.CXM.CXM-A.CTX	5	5 (5)	0	0	0	0	5
B	AMP.TMP/SXT.CXM.CXM-A.CTX	1	3 (3)	2	0	0	0	1
	AMP.TMP/SXT.CXM.CXM-A.CTX.CAZ	1						
	AMP.TMP/SXT.CXM.CXM-A.CTX.CAZ.AMC.GEN.CIP	1						
C	AMP.CXM.CXM-A.CTX.TMP/SXT.GEN.AMC	2	3 (3)	0	1	0	0	2
	AMP.CXM.CXM-A.CTX.CIP.TMP/SXT	1						
D	AMP.TMP/SXT.CXM.CXM-A.CTX	10	12 (13)	0	0	3	0	7
	AMP.TMP/SXT.CXM.CXM-A.CTX.CAZ	1						
	AMP.CXM.CXM-A.CTX.CIP.TMP/SXT.AMC	1						
E	AMP.TMP/SXT.CXM.CXM-A.CTX	2	4 (4)	1	1	0	0	2
	AMP.TMP/SXT.CXM.CXM-A.CTX.CAZ	1						
	AMP.TMP/SXT.CXM.CXM-A.CTX.CAZ.FEP	1						
F	AMP.TMP/SXT.CXM.CXM-A.CTX	4	4 (4)	0	0	0	0	4
G	AMP.TMP/SXT.CXM.CXM-A.CTX.CAZ	2	3 (3)	3	0	0	0	0
	AMP.CXM.CXM-A.CTX.CAZ.CIP.TMP/SXT.AMC.TZP	1						
H	AMP.CXM.CXM-A.CTX.TMP/SXT.CAZ.FEP.AN.GEN.CS	1	4 (4)	2	1	0	0	1
	AMP.TMP/SXT.CXM.CXM-A.CTX.AMC.TZP.CAZ	1						
	AMP.TMP/SXT.CXM.CXM-A.CTX.CAZ.AMC.FEP.GEN.CIP	1						
	AMP.AMC.CXM.CXM-A.CTX.CIP	1						
I	AMP.TMP/SXT.CXM.CXM-A.CTX	2	18 (19)	13	2	2	1	0
	AMP.TMP/SXT.CXM.CXM-A.CTX.CAZ	2						
	AMP.TMP/SXT.CXM.CXM-A.CTX.CAZ.FEP	1						
	AMP.TMP/SXT.CXM.CXM-A.CTX.CAZ.GEN.CIP	1						
	AMP.TMP/SXT.CXM.CXM-A.CTX.CAZ.AMC.GEN.CIP	1						
	AMP.TMP/SXT.CXM.CXM-A.CTX.AMC.TZP.CAZ	5						
	AM.CXM.CXM-A.CTX.FEP.TMP/SXT	1						
	AMP.CXM.CXM-A.CTX.CAZ	2						

	AMP.CXM.CXM-A.CTX.CIP.TMP/SXT.AMC	2						
	AMP.CXM.CXM-A.CTX.GEN.TMP/SXT	1						
**J**	AMP.TMP/SXT.CXM.CXM-A.CTX	1	8 (9)	7	0	1	0	0
	AMP.TMP/SXT.CXM.CXM-A.CTX.CAZ	3						
	AMP.TMP/SXT.CXM.CXM-A.CTX.CAZ.FEP	1						
	AMP.TMP/SXT.CXM.CXM-A.CTX.CAZ.GEN.CIP	1						
	AMP.TMP/SXT.CXM.CXM-A.CTX.AMC.TZP.CAZ	1						
	AMP.CXM.CXM-A.CTX.CIP.TMP/SXT.AMC	1						
**K**	AMP.TMP/SXT.CXM.CXM-A.CTX	1	12 (13)	9	3	0	0	0
	AMP.TMP/SXT.CXM.CXM-A.CTX.CAZ	1						
	AMP.TMP/SXT.CXM.CXM-A.CTX.CAZ.AMC	2						
	AMP.TMP/SXT.CXM.CXM-A.CTX.CAZ.AMC.GEN.CIP	1						
	AMP.TMP/SXT.CXM.CXM-A.CTX.AMC.TZP.CAZ	3						
	AMP.CXM.CXM-A.CAZ.CS	1						
	AMP.TMP/SXT.CXM.CXM-A.CTX.CAZ.AMC.TZP.FOX.FEP.GEN.CIP	1						
	AMP.CXM.CXM-A.CTX.CAZ.CIP.GEN.TMP/SXT.TZP.FEP	1						
	AMP.CXM.CXM-A.CTX.CAZ.CIP.GEN.TMP/SXT.TZP	1						
**L**	AMP.TMP/SXT.CXM.CXM-A.CTX	4	8 (9)	8	0	0	0	0
	AMP.TMP/SXT.CXM.CXM-A.CTX.CAZ	2						
	AMP.CXM.CXM-A.CTX.CIP.TMP/SXT	1						
	AMP.CXM.CXM-A.CTX.GEN.TMP/SXT	1						
**M**	AMP.TMP/SXT.CXM.CXM-A.CTX	4	7 (9)	0	0	0	4	4
	AMP.CXM.CXM-A.CTX	1						
	AMP.CXM.CXM-A.CTX.TMP/SXT.GEN	1						
	AMP.CXM.CXM-A.CTX.TMP/SXT.GEN.AMC	1						
**N**	AMP.TMP/SXT.CXM.CXM-A.CTX.CAZ	1	3 (3)	3	0	0	0	0
	AMP.TMP/SXT.CXM.CXM-A.CTX.CAZ.AMC	1						
	AMP.CXM.CXM-A.CTX.CAZ.FEP.GEN.TMP/SXT	1						
**Grand Total**		**93**	**93 (100)**	48	8	6	5	26

AMP: Ampicillin; AMC: Amoxicillin-clavulanate; AK: Amikacin; CXM: Cefuroxime; CXM-A: Cefuroxime-axetyl; CTX: Cefotaxime; CAZ: Ceftazidime; CS: Colistin; CIP: Ciprofloxacin; FEP: Cefepime; FOX: Cefoxitin; GM: Gentamicin; TMP/SXT: Trimethoprim-sulfamethoxazole; TZP: Piperacillin-tazobactam
